# Supplementary material for: Engineering new-to-nature biochemical conversions by combining fermentative metabolism with respiratory modules
Source: Nat Commun. 2024 Aug 7;15:6725. doi: 10.1038/s41467-024-51029-x (PMC11306353; doi:10.1038/s41467-024-51029-x)
Supplement: Supplementary file 1 — Supplementary information [file 41467_2024_51029_MOESM1_ESM.pdf]

Engineering new-to-nature biochemical conversions by combining fermentative metabolism with respiratory modules

Schulz-Mirbach *et al.*

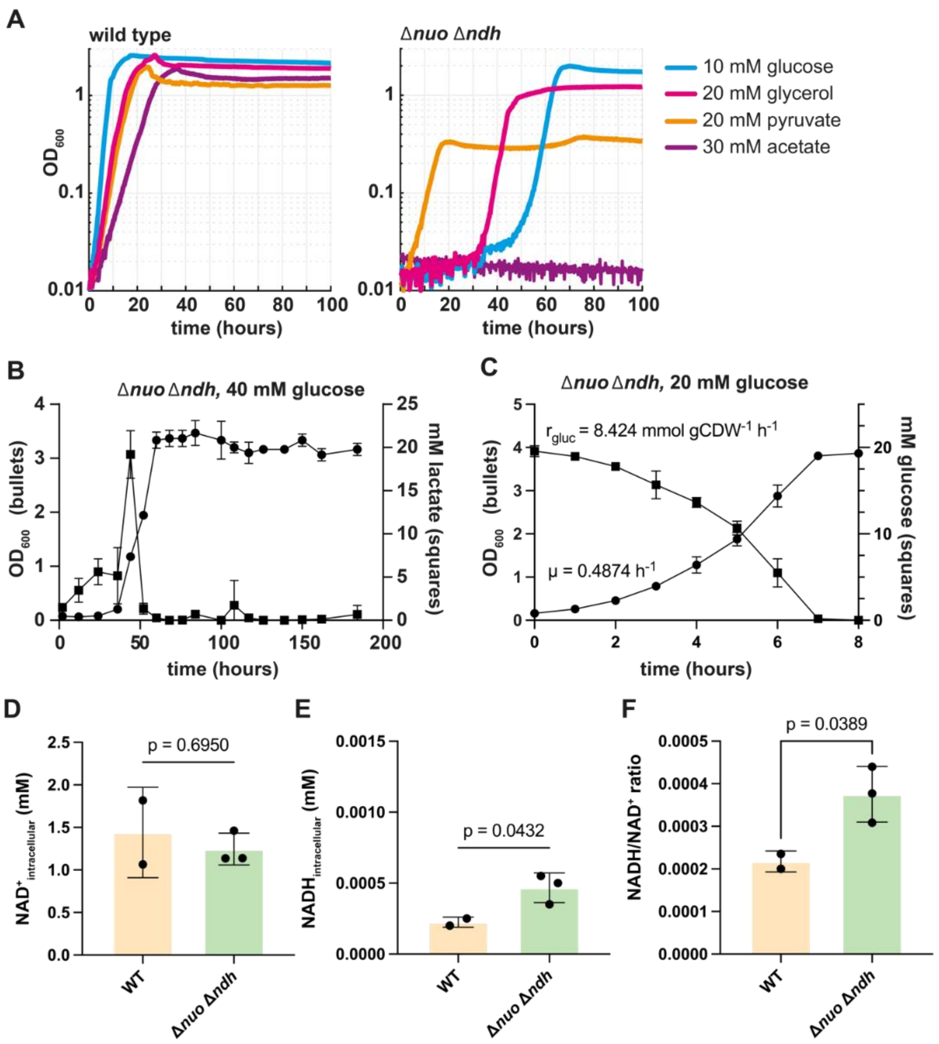

Supplementary Figure 1. Aerobic characterization of an NAD(P)H dehydrogenase deficient strain. A) Comparison of growth phenotype on different carbon sources of an NAD(P)H-dehydrogenase deficient strain (Δ*nuoEFG* Δ*ndh*) with a wild-type. On acetate, growth is abolished for the Δ*nuoEFG* Δ*ndh* strain while growth on the other carbon sources exhibits an extended lag phase and lower biomass yields. **B)** Lactate production (squares) and OD_{600} (bullets) was measured during the aerobic cultivation of the Δ*nuoEFG* Δ*ndh* strain on 40 mM glucose. Mean values of triplicate measurements (n=3) are shown with standard deviation, an outlier for the lactate production at t = 44 h was excluded. **C)** Growth of the Δ*nuoEFG* Δ*ndh* strain on 20 mM glucose. Growth rate und glucose consumption were determined in technical triplicates, the mean of those is shown with standard deviation. **D)** Intracellular NAD^+ concentrations in the WT and the Δ*nuoEFG* Δ*ndh* strain grown with 20 mM glucose. Comparisons were done using an unpaired *t*-test. Concentrations were determined in triplicates, mean values are shown with standard deviation (n=3). An outlier (0.013 mM) was omitted for the wild type. **E)** Estimated intracellular NADH concentrations in the WT and the Δ*nuoEFG* Δ*ndh* strain grown with 20 mM glucose. Comparisons were done using an unpaired *t*-test (n=3). Mean values are shown with standard deviation. Low intracellular NADH concentrations are likely due to a suboptimal extraction method. **F)** Ratios of intracellular NADH over intracellular NAD^+ in the WT and the Δ*nuoEFG* Δ*ndh* strain grown with 20 mM glucose. Mean values are shown with standard deviation. Comparisons were done using an unpaired *t*-test (n=3). Source data are provided as a Source Data file.

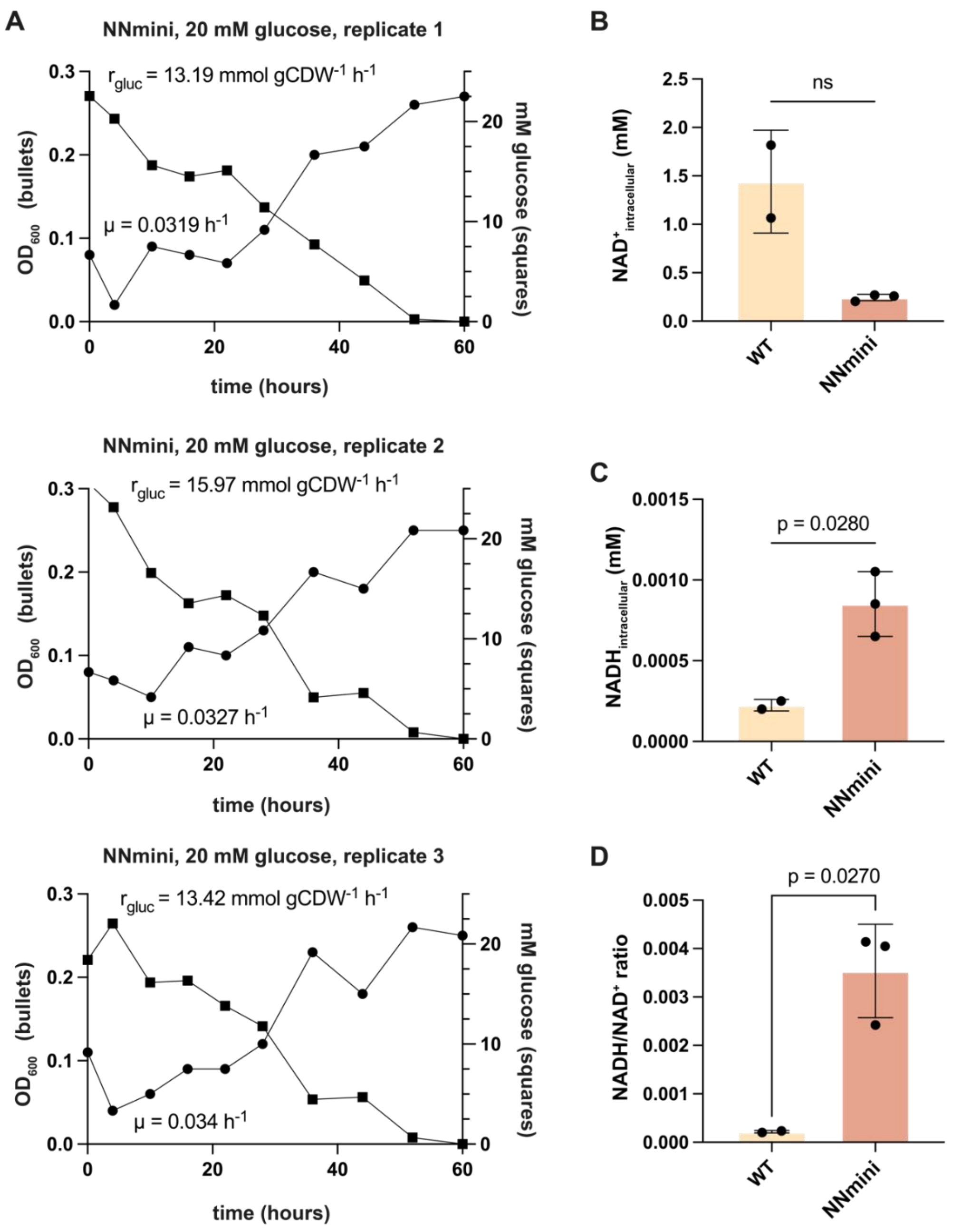

Supplementary Figure 2. Characterisation of the aerobically growing NNmini strain. A) Growth of the NNmini strain on 20 mM glucose. Growth rate und glucose consumption were determined in biological triplicates. **B)** Intracellular NAD^+ concentrations in the WT and the NNmini strain grown with 20 mM glucose. Comparisons were done using an unpaired *t*-test. Concentrations were determined in triplicates (n=3). Mean values are shown with standard deviation. An outlier (0.013 mM) was omitted for the wild type. **C)** Estimated intracellular NADH concentrations in the WT and the NNmini strain grown with 20 mM glucose. Comparisons were done using an unpaired *t*-test (n=3). Mean values are shown with standard deviation. Low intracellular NADH concentrations are likely due to a suboptimal extraction method. **D)** Ratios of intracellular NADH over intracellular NAD^+ in the WT and the NNmini strain grown with 20 mM glucose. Comparisons were done using an unpaired *t*-test (n=3). Mean values are shown with standard deviation. Source data are provided as a Source Data file.

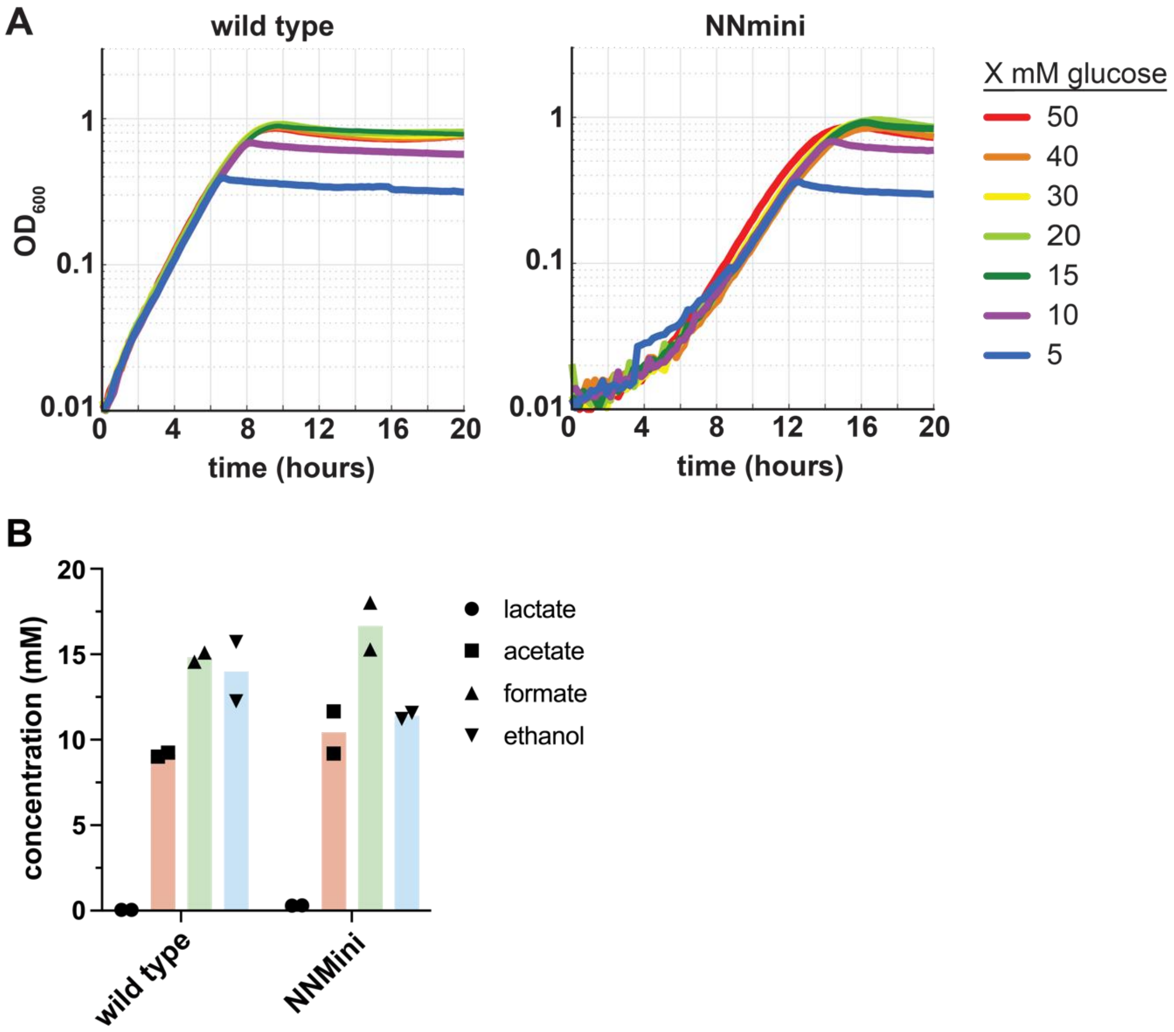

Supplementary Figure 3. Anaerobic characterization of the NNmini strain compared to the wild type. A) Anaerobic growth of the NNmini strain and a wild type in minimal medium supplemented with different glucose concentrations. **B)** Comparison of fermentation products from wild type and NNmini grown anaerobically on 10 mM glucose. The mean of duplicate measurements and individual datapoints are shown. Source data are provided as a Source Data file.

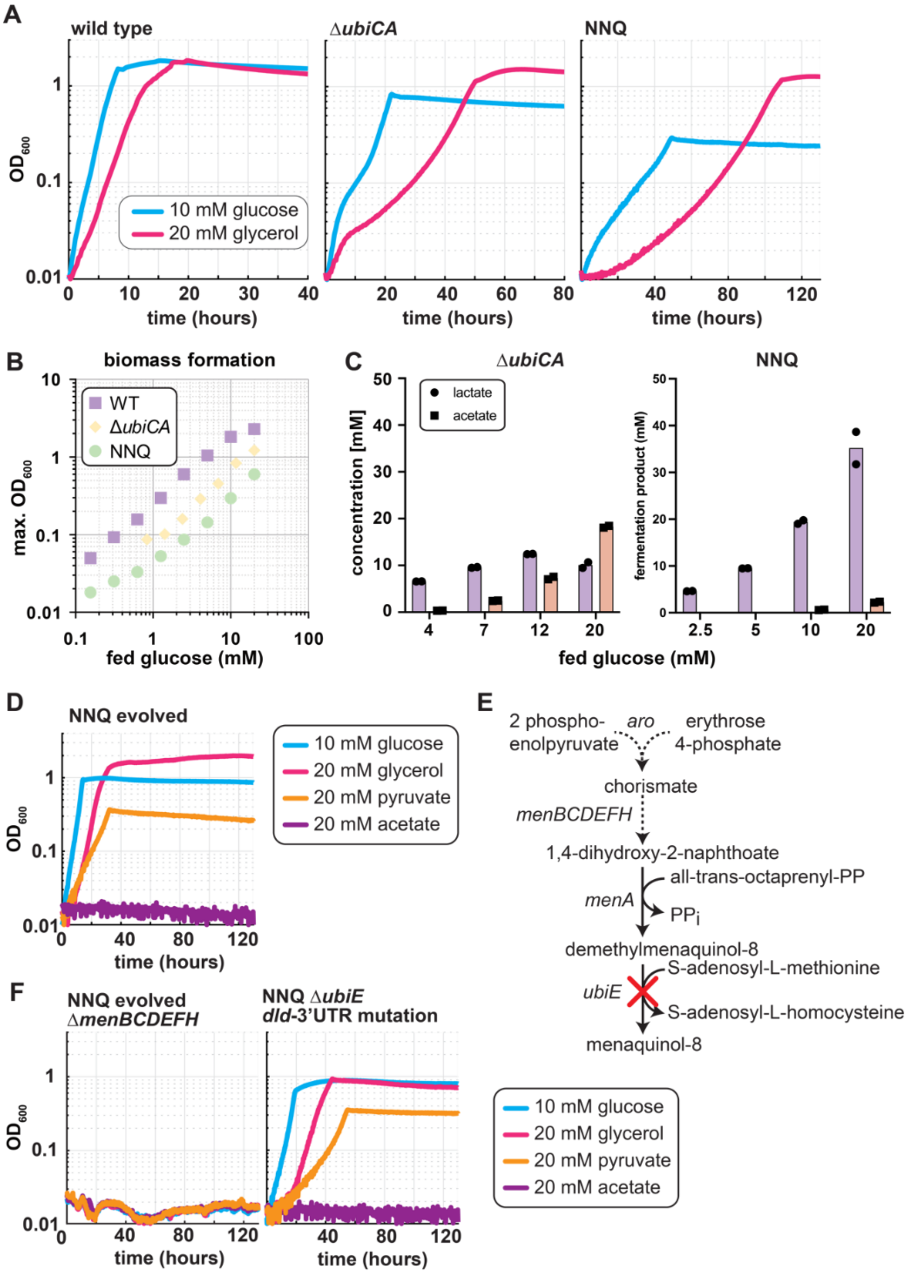

Supplementary Figure 4. Aerobic characterization of the Δ*ubiCA* and the NNQ strain. A) Growth of a wild-type control strain (right), the Δ*ubiCA* strain (middle) and) the NNQ strain (left) on 10 mM glucose (blue) or 20 mM glycerol (pink). **B)** Both NNQ (green bullets) and Δ*ubiCA* strain (yellow diamonds) grow to lower biomass yields, indicated by maximum OD_{600}, than the wild type (purple squares) with glucose as sole carbon source. Mean values of duplicate measurements are plotted. **C)** Fermentation products detected in culture supernatants from the Δ*ubiCA* and the NNQ strain grown on glucose. Mean values of duplicate measurements and individual datapoints are shown. **D)** The evolved NNQ strain grows to higher biomass yields with glucose (blue) and glycerol (pink). **E)** Schematic view of ubiquinone synthesis and the disruption of *ubiE*, which could cause demethylmenaquinone accumulation in the evolved NNQ strain. **F)** A knockout of the menaquinone biosynthesis genes is lethal for the evolved NNQ strain. Re-engineering of the discovered mutations in the evolved NNQ strain allow respiratory growth of an unevolved NNQ strain. Source data are provided as a Source Data file.

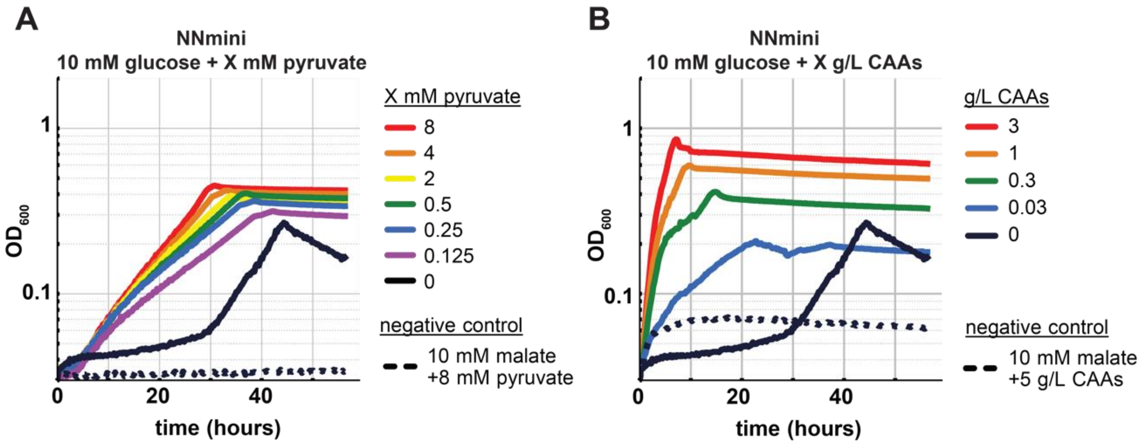

Supplementary Figure 5. Comparison of pyruvate and CAA supplementation to NNmini strain growing aerobically on glucose A) Supplementation of a pyruvate gradient to NNmini strain growing aerobically on 10 mM glucose. **B)** Supplementation of a casamino acid (CAA) gradient to NNmini strain growing aerobically on 10 mM glucose. Source data are provided as a Source Data file.

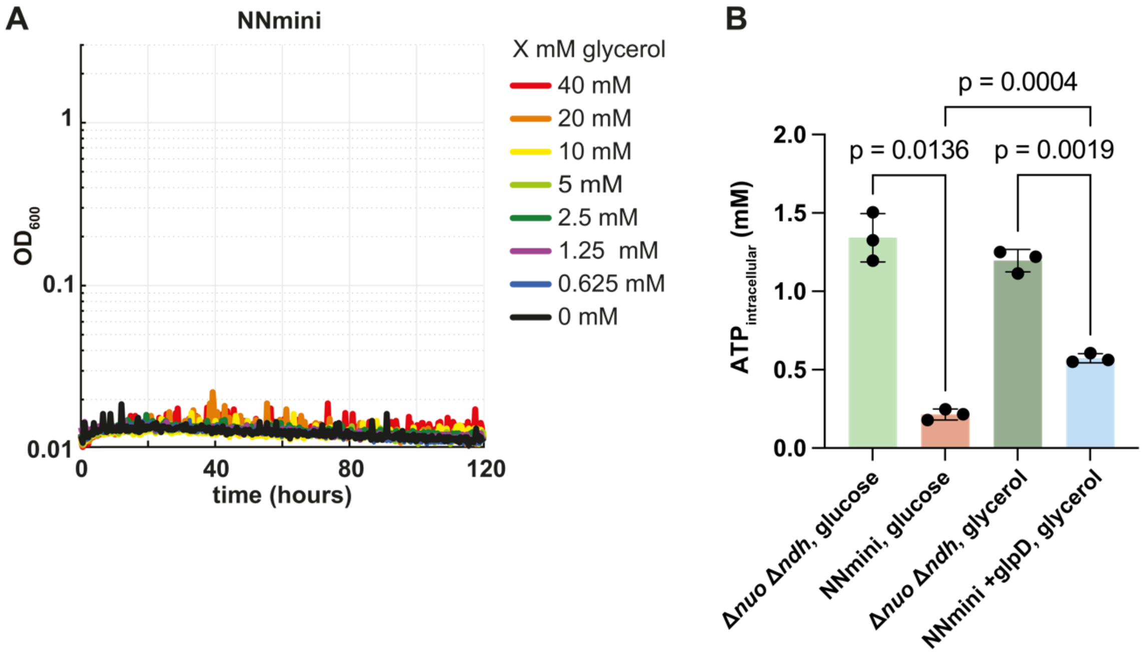

Supplementary Figure 6. Glycerol utilization requires presence of the quinone dependent GlpD which allows increased ATP synthesis. A) The NNmini strain is unable to grow on glycerol. Glycerol concentrations are indicated by the legend to the right. **B)** Intracellular ATP concentrations from Δ*nuoEFG* Δ*ndh*, and NNMini or NNMini +*glpD* grown on 20 mM glucose or 40 mM glycerol, respectively. Pairwise comparisons were done using an unpaired *t*-test. Concentrations were determined in triplicates (n=3). Mean values are shown with standard deviation. Source data are provided as a Source Data file.

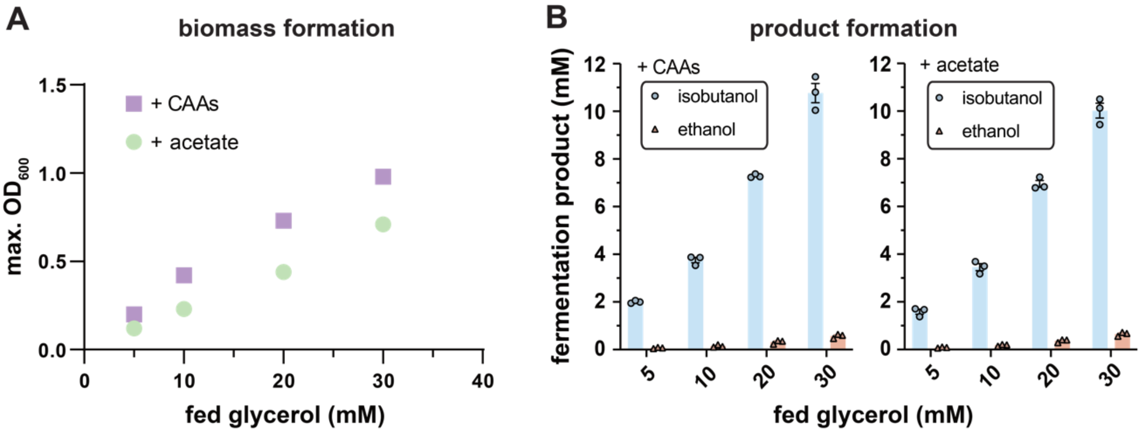

Supplementary Figure 7. Growth of the NNmini +glpD Δ*ldhA* +pIBA with acetate or casamino acid supplementation. A) Biomass yields in M9 medium with glycerol and 10 mM acetate (green) or 5 g/L casamino acid (violet) supplementation determined from tube cultivations (n = 1). **B)** Isobutanol (light blue) and ethanol (orange) yields of the NNMini +glpD Δ*ldhA* +pIBA grown with glycerol and 5 g/L casamino acids (left) or 10 mM acetate (right) (n=3). Mean values are shown with standard deviation. Source data are provided as a Source Data file.

Supplementary Note 1. Manual curation of identified quinone-reducing reactions

In total, 17 quinone reducing reactions were initially identified in the iML1515 model (Supplementary Data File 1). After cross-referencing these with the database EcoCyc [1], we decided to excluded the following reactions from the pool of deletion candidates: ASPO3, DHORD2, DSBAO1, FDH4pp, HXD1pp, GLCDpp. ASPO3, the L-aspartate oxidase, was annotated to operate with oxygen or fumarate, but not with quinones as electron acceptor [2]. High-throughput knockout library characterizations revealed the essentiality of the dihydroorotate dehydrogenase (DHORD2) for *E. coli* growth with glucose or glycerol as carbon and energy sources [3,4]. Since such growth was essential for the NNmini target applications, we decided not to delete this candidate. Similarly, the thiol-disulfide oxidoreductase DsbA (DSBAO1) was annotated to be essential for *E. coli* growth in anaerobic conditions and was therefore omitted from the candidate pool [5]. The formate dehydrogenase (FDH4pp) was not included because formate formation was not expected in aerobic conditions due to the oxygen sensitivity of pyruvate-formate lyase [6]. Similarly, hydrogenase 1 (HYD1pp) was ruled out as candidate because expression of the *hya* operon is only induced in anaerobic conditions [7]. Finally, the membrane-bound glucose dehydrogenase (GLCDpp) requires pyrroloquinoline quinone as cofactor, which *E. coli* is unable to synthesize [8,9]. Three reactions and their associated genes were manually added to the list of deletion candidates: *lhgO*, encoding an L-2-hydroxyglutarate dehydrogenase, and *dadA*, a D-amino acid dehydrogenase, because both are annotated to utilize quinones in EcoCyc [1]. The acyl-CoA dehydrogenase encoded by *fadE* is annotated to be FAD^+ and not quinone-dependent. However, it was deleted since it oxidizes a wide array of fatty acyl-CoA esters with FAD^+ as an electron acceptor and was speculated to re-oxidize $FADH_2$ by transferring electrons to the ETC [10]. In addition to the ubiquinone-dependent reactions, we also identified 6 demethymenaquinone (DMMQ)- and 12 menaquinone (MQ)-dependent reactions in the iML1515 model. However, both of these quinone-species are not expected to play a role under aerobic conditions[11] – therefore, the respective enzymes were therefore not considered as deletion targets.

Supplementary Note 2. Deleting (ubi)quinone biosynthesis together with NADH dehydrogenases results in unstable fermentative growth

To investigate whether the aerobic fermentative phenotype of the NNmini strain could be achieved with less genetic modifications, we deleted the ubiquinone biosynthesis (encoded by *ubiCA*) both alone and in the Δ*nuoEFG* Δ*ndh* background. We engineered the corresponding strains and called the Δ*nuoEFG* Δ*ndh* Δ*ubiCA* strain "NNQ" where "NN" denotes the deletion of the major NADH dehydrogenases and "Q" denotes the deletion of ubiquinone biosynthesis.

Both the Δ*ubiCA* and NNQ strains showed slower growth rates on glucose and glycerol than observed for the wild-type (Supplementary Fig. 4A). Biomass yields of the deletion mutants were lower than those obtained for the wild-type (Supplementary Fig. 4B), which hinted at an aerobic fermentative phenotype. Both strains produced lactate and acetate from glucose (Supplementary Fig. 4C), which is in line with previous characterizations of *ubiA* and *ubiCA* deletion strains [11,12]. Notably, the NNQ strain converted glucose into lactate and only residual acetate at a stoichiometry that is close to the theoretical maximal fermentation product yield for *E. coli* (Supplementary Fig. 4C). For both strains, the secretion of acetate indicates that the strains are still able to oxidize pyruvate. Since experiments were carried out in the presence of oxygen and no formate was detected, NNQ and Δ*ubiCA* strains seem to possess some electron sinks other than pyruvate-formate lyase which allow the secretion of acetate as an unbalanced fermentative product. Moreover, we found that upon prolonged cultivation, the NNQ strain grew much

faster and to higher cell densities than previously observed in our experiments (Supplementary Fig. 4D). Genome sequencing of independently selected isolates revealed that they shared mutations in the *dld* promoter region and missense mutations in the *ubiE* gene (Supplementary Data File 2). The mutation in the genomic region upstream of the dld gene possibly results in a slight increase of its translation initiation (translation initiation rate = 2649 compared to 1824 in the wild-type, calculated with RBS Calculator in predict mode)[13]. This might facilitate uptake of previously excreted lactate and thereby together with the NADH-dependent lactate dehydrogenase result in a mini cycle for NAD^+ regeneration (Figure 2). Additionally, the mutations in the ubiE region likely lead to the inactivation of UbiE, which could result in an accumulation of demethylmenaquinone (DMK) (Supplementary Fig. 4E). Indeed, previous works described DMK accumulation upon UbiE inactivation [14]. We hypothesized that DMK accumulation could restore cellular respiration by allowing ubiquinone-dependent dehydrogenases to use DMK as an electron acceptor. To verify these hypothesis, we reverse-engineered these mutations (by deleting *ubiE* and inserting the mutation in the 3'-UTR of the *dld* gene) in a naïve NNQ strain and demonstrated an improved growth phenotype. Furthermore, we deleted the menaquinone biosynthesis (Δ*menBCDEFH*) in the evolved NNQ strain, which coherently abolished growth on all tested carbon sources (Supplementary Fig. 4F). Notably, we reasoned that such replacements should not occur in the metabolic context of the NNmini strain, since there all quinone dependent dehydrogenases were deleted.

Taken together, these findings demonstrate that the NNQ strain exhibited only an unstable aerobic fermentative phenotype. This limits biotechnological applications especially for long time cultivations. Furthermore, in the NNQ strain, it is not possible to use oxygen for single quinone-dependent reactions like in the NNmini strain. It can therefore not be used to perform re-balanced fermentations in a controlled manner.

Supplementary references

1. Keseler, I. M. *et al.* The EcoCyc database: reflecting new knowledge about *Escherichia coli* K-12. *Nucleic Acids Res.* **45**, D543–D550 (2017).
2. Chow, C., Hegde, S. & Blanchard, J. S. Mechanistic characterization of *Escherichia coli* l-aspartate oxidase from kinetic isotope effects. *Biochemistry* **56**, 4044–4052 (2017).
3. Patrick, W. M., Quandt, E. M., Swartzlander, D. B. & Matsumura, I. Multicopy suppression underpins metabolic evolvability. *Mol. Biol. Evol.* **24**, 2716–2722 (2007).
4. Joyce, A. R. *et al.* Experimental and computational assessment of conditionally essential genes in *Escherichia coli*. *J. Bacteriol.* **188**, 8259–8271 (2006).
5. Meehan, B. M., Landeta, C., Boyd, D. & Beckwith, J. The Disulfide Bond Formation Pathway Is Essential for Anaerobic Growth of *Escherichia coli*. *J. Bacteriol.* **199**, e00120-17 (2017).
6. Zhang, W., Wong, K. K., Magliozzo, R. S. & Kozarich, J. W. Inactivation of pyruvate formate-lyase by dioxygen: defining the mechanistic interplay of glycine 734 and cysteine 419 by rapid freeze-quench EPR. *Biochemistry* **40**, 4123–4130 (2001).
7. Brøndsted, L. & Atlung, T. Anaerobic regulation of the hydrogenase 1 (hya) operon of *Escherichia coli*. *J. Bacteriol.* **176**, 5423–5428 (1994).
8. Kobayashi, K., Mustafa, G., Tagawa, S. & Yamada, M. Transient formation of a neutral ubisemiquinone radical and subsequent intramolecular electron transfer to pyrroloquinoline quinone in the *Escherichia coli* membrane-integrated glucose dehydrogenase. *Biochemistry* **44**, 13567–13572 (2005).
9. Matsushita, K. *et al. Escherichia coli* is unable to produce pyrroloquinoline quinone (PQQ). *Microbiol. Read. Engl.* **143 (Pt 10)**, 3149–3156 (1997).
10. Campbell, J. W. & Cronan, J. E. The enigmatic *Escherichia coli* fadE gene is yafH. *J. Bacteriol.* **184**, 3759–3764 (2002).
11. Wallace, B. J. & Young, I. G. Role of quinones in electron transport to oxygen and nitrate in *Escherichia coli*. Studies with a ubiA- menA- double quinone mutant. *BBA - Bioenerg.* **461**, 84–100

(1977).
12. Zhu, J., Sánchez, A., Bennett, G. N. & San, K. Y. Manipulating respiratory levels in *Escherichia coli* for aerobic formation of reduced chemical products. *Metab. Eng.* **13**, 704–712 (2011).
13. LaFleur, T. L., Hossain, A. & Salis, H. M. Automated model-predictive design of synthetic promoters to control transcriptional profiles in bacteria. *Nat. Commun.* **13**, 5159 (2022).
14. Wissenbach, U., Ternes, D. & Unden, G. An *Escherichia coli* mutant containing only demethylmenaquinone, but no menaquinone: effects on fumarate, dimethylsulfoxide, trimethylamine N-oxide and nitrate respiration. *Arch. Microbiol.* **158**, 68–73 (1992).
