# Supplementary material for: Engineering new-to-nature biochemical conversions by combining fermentative metabolism with respiratory modules
Source: Nat Commun. 2024 Aug 7;15:6725. doi: 10.1038/s41467-024-51029-x (PMC11306353; doi:10.1038/s41467-024-51029-x)
Supplement: Supplementary file 3 — Description of Additional Supplementary Files [file 41467_2024_51029_MOESM3_ESM.pdf]

Description of Additional Supplementary Files

File Name: Supplementary Data 1
Description: Oligonucleotide primers used. 'KO' primers were used to amplify the Km knockout cassette from pKD4 with 50 bp gene-specific upstream and downstream sequences [Datsenko & Wanner et al.]. To verify gene replacement by kanamycin resistance cassette and cassette removal by flippase, 'KO-Ver'-primers (knockout-verification) were used. Internal primers were used to verify successful removal of the gene from the genome.

File Name: Supplementary Data 2
Description: List of reactions in the iML1515 model reducing ubiquinone or potentially reducing ubiquinone via FAD^+. Reactions are described with reaction ID, reaction name, reaction equation and gene rule. Furthermore, details about metabolite identifiers are given explaining the metabolite full name, compartment localisation and chemical formula.

File Name: Supplementary Data 3
Description: Description of whole genome sequencing results from NNmini, NNQ and evolved NNQ strains. Mutations are described with their position on the genome, predicted mutation, and the corresponding annotation, gene and gene description.
